# Effect of Pore Size and Porosity on the Biomechanical Properties and Cytocompatibility of Porous NiTi Alloys

**DOI:** 10.1371/journal.pone.0128138

**Published:** 2015-06-05

**Authors:** Yu-Tao Jian, Yue Yang, Tian Tian, Clark Stanford, Xin-Ping Zhang, Ke Zhao

**Affiliations:** 1 Institute of Stomatological Research, Guangdong Provincial Key Laboratory of Stomatology, Sun Yat-sen University, Guangzhou, Guangdong, The Peoples Republic of China; 2 Department of Prosthodontics, Guanghua School of Stomatology, Sun Yat-sen University, Guangdong Provincial Key Laboratory of Stomatology, Guangzhou, Guangdong, The Peoples Republic of China; 3 Dows Institute for Dental Research College of Dentistry, University of Iowa, Iowa city, Iowa, United States of America; 4 Department of Metallic Materials Science and Engineering, School of Materials Science and Engineering, South China University of Technology, Guangzhou, Guangdong, The Peoples Republic of China; University of Akron, UNITED STATES

## Abstract

Five types of porous Nickel-Titanium (NiTi) alloy samples of different porosities and pore sizes were fabricated. According to compressive and fracture strengths, three groups of porous NiTi alloy samples underwent further cytocompatibility experiments. Porous NiTi alloys exhibited a lower Young’s modulus (2.0 GPa ~ 0.8 GPa). Both compressive strength (108.8 MPa ~ 56.2 MPa) and fracture strength (64.6 MPa ~ 41.6 MPa) decreased gradually with increasing mean pore size (MPS). Cells grew and spread well on all porous NiTi alloy samples. Cells attached more strongly on control group and blank group than on all porous NiTi alloy samples (*p* < 0.05). Cell adhesion on porous NiTi alloys was correlated negatively to MPS (277.2 μm ~ 566.5 μm; *p* < 0.05). More cells proliferated on control group and blank group than on all porous NiTi alloy samples (*p* < 0.05). Cellular ALP activity on all porous NiTi alloy samples was higher than on control group and blank group (*p* < 0.05). The porous NiTi alloys with optimized pore size could be a potential orthopedic material.

## Introduction

Commercially pure Titanium (cpTi) and its alloys have been shown to be a good biomaterial for orthopedic and dental applications [1,2]. In general, the Titanium (Ti) and its alloys used for orthopedic are mostly dense materials. In comparison to cancellous and cortical bone with Young’s modulus ranging from 0.5 GPa to 20 GPa [3], the modulus of dense Ti alloys reaches about 114 GPa. Such a distinguishingly high modulus value may create a stress-shielding effect [4] at the bone tissue-implant interface and lead to loosening of the implant. Further, the solid structure of the implant shows a low ability to induce implant fixation and regeneration of bone tissue [5].

To maintain the original alloy composition, the introduction of porous structure is deemed to be a possible way to reduce the elastic modulus of metal materials [6]. A porous structure may also provide new abilities for the tissue ingrowth and vascularization of bone [7]. Although the application of some porous bone-substitute materials such as porous ceramics and natural polymers is limited to the mechanical properties [8], the introduction of pores into the dense alloys sheds light on the overcoming the disadvantages of dense cpTi and meanwhile regulating its mechanical property. Porous Nickel–Titanium (NiTi) alloys are excellent candidates for a wide range of biomedical applications. A study has demonstrated that new bone penetrated deeper into porous NiTi than porous Ti [4] suggesting that porous NiTi implants might have better osteointegration and osteoconductivity than porous Ti due to similar super-elasticity to natural bones.

The elastic modulus of a porous material is mainly dependent upon porosity. The structure features of porous metallic scaffolds, like porosity and pore size, are crucial to their mechanical and biological properties, and may determine their performance after insertion into the bone defect area [9]. However, the effect of pore size on the mechanical behavior is still controversial in the literature [9,10]. A previous study showed that porous Ti with smaller pore size has higher strength than that with larger pore size [11]. Yet, this enhanced strength has to be balanced with an inability for bone ingrowth. Another study has indicated the compressive stress-strain curve ascends constantly with enlarging pore size [12]. Some studies have shown that a porous morphology with pore size larger than 150 μm provides a favorable environment for the ingrowth of natural bone [13,14], whereas a study has suggested that pore size between 25 μm and 200 μm has the most positive effect on the growth of human osteoblastic cell [9]. Moreover, these studies with divergent results on the pore size do not study porosity in each group at all.

Porosity and pore size are the two main independent but correlated factors of porous materials. Here we present the results of a preliminary study into the effect of pore size and porosity on the mechanical and biological properties of porous NiTi alloys for possible orthopedic applications, and seeking the optimal pore size in terms of the cytocompartibility.

## Materials and Methods

### Preparation and characterization of porous NiTi

Elemental Ti powder (size 50 μm ~ 74 μm, purity 99.3 wt.%) and Nickel (Ni) powder (size 74 μm, purity 99.5 wt.%), with a nominal atomic ratio of 50.8 at.% Ni to 49.2 at.% Ti, were blended with high-purity NH_4_HCO_3_ powder (size 0 μm ~ 900 μm, purity 99.9%), which was used as temporary space-holder and porosity regulator. Porous NiTi alloy samples were fabricated as described in a previous work [15]. Five groups of porous NiTi alloy samples (A-E) were designed by using NH_4_HCO_3_ powder of different particle sizes (Table 1). Commercially pure titanium (cpTi) was treated as control (group F).

**Table 1:** Nominal composition of five groups of porous NiTi alloy samples.

Group	Mixed metal powder (80 wt.%)		NH_4_HCO_3_ (20 wt.%)
	Ni (at.%)	Ti (at.%)	NH_4_HCO_3_ size (μm)
A	50.8	49.2	600 ~ 900
B	50.8	49.2	450 ~ 600
C	50.8	49.2	300 ~ 450
D	50.8	49.2	200 ~ 300
E	50.8	49.2	50 ~ 200

at.%: nominal atomic percent; wt.%: weight percent.

Microstructure, pore morphology and pore size of the fabricated porous NiTi alloy samples were visualized by means of metallographic microscopy (Olympus, Tokyo, Japan) and analyzed by a software package Microsystem Imaging Solutions Analysis (Leica, Wetzlar, Germany) [15,16]. The general porosity of porous NiTi alloy samples, *p*, was calculated by the following equation:

$$p\;=\;(1-\frac{\rho }{\rho 0})\times 100\%$$ (1)

where *ρ* is the apparent density of porous NiTi alloys (measured by dividing the weight by the volume of the sample) and *ρ*_0_ is the theoretical density of the corresponding dense NiTi alloys (6.45 g/cm^3^).

### Mechanical properties of porous NiTi

The cylindrical specimens with a diameter of 4 mm and a height of 12 mm were used to evaluate the compressive modulus and compressive strength of porous NiTi alloy samples with different pore sizes and porosities. The compression tests were conducted using a computer-controlled universal testing machine (AG-X 100 kN, Shimadzu, Japan) at a crosshead speed of 2.4 mm/min. Rectangular specimens with the geometry of 3 × 4 × 25 (thickness × width × length, mm^3^) were used to carry out three-point bending tests with a loading rate of 0.5 mm/min and thus estimate the bending strength of porous NiTi alloy samples. 5 samples in each of five groups were used for obtaining the average value of mechanical properties.

### Cytocompatibility of porous NiTi

Specimens with a diameter of 10 mm and a height of 2 mm cut from as-fabricated porous NiTi alloy samples were used to test the cytocompatibility. Experiments were undertaken in triplicate, and triplicate discs were used in each experiment. The cytocompatibility of specimens was examined through a series of tests as cell adhesion, cell proliferation, alkaline phosphatase (ALP) test, and the growth morphology was visualized using scanning electron microscope (SEM, Quanta 200, FEI, Netherlands).

#### Cell culture

It is well known that different cell lines can respond in different patterns to the same surface topography [17]. We chose the human osteoblast cells hFOB 1.19 since the cell line has been widely used as a model of normal osteoblast differentiation [18,19].

The hFOB 1.19 cells (CRL-11372; American Type Culture Collection, Manassas, US) were cultured in 90% DMEM Nutrient Mixture F-12 Ham (Sigma-Aldrich, St Louis, US) with 2.5 mM L-glutamine and 15 mM HEPES supplemented with 0.3 mg/mL G-418 (Merck, Whitehouse Station, US) and 10% FBS (HyClone, Thermo, Germany). Cells were grown in 25 cm^2^ plastic culture flasks and incubated at 33.5°C. At about 90% confluence cells were washed thrice with PBS and harvested using 0.25% trypsin-0.53 mM EDTA (Gibco, Grand Island, US) at 37°C for 5 min. Cells were further centrifuged at 1,763 × *g* for 5 min at 37°C, and subcultured in a 1:3 ratio.

#### Cell morphology

hFOB 1.19 cells were seeded at 5×10^4^ cells/ml on surface of disc samples, and incubated for 3 days at 33.5°C in serum-containing media. Discs incubated without cells were as negative controls. Cells growing on glass coverslips were used as controls for cell growth. Samples were washed carefully with PBS and fixed overnight in 4% glutaraldehyde buffered in PBS at 4°C. After washing thrice in PBS, the samples were dehydrated in a graded series of ethanol solutions (10%- 100%) for 10 min each, followed by drying in a Critical Point Dryer (Hitachi, Tokyo, Japan). Samples were mounted on stubs and sputtered with gold using an Ion Coater (Eiko, Tokyo, Japan), and then visualized using a SEM at 15 kV to 20 kV.

#### Cell adhesion and proliferation assay

hFOB 1.19 cells were seeded at 2×10^5^ cells/ml on all groups C ~ E discs and cpTi, cells cultured directly on polystyrene plate as blank for cell growth. Cells were incubated in serum-containing media at 33.5°C in 5% CO_2_ for 30, 60, 120, 180 and 240 min for cell adhesion assay, and 1, 3, 5, 7 and 9 days for cell proliferation assay, respectively. Viable cell numbers were evaluated at appropriate time intervals using the MTS assay (Promega, Madison, US) according to manufacturer instructions. The optical density (OD) value was read at 490 nm using an ELISA plate reader.

#### ALP activity testing

ALP activity is an early marker for osteoblast differentiation. Cell ALP activity was assayed using a commercial kit (Jiancheng, Nanjing, China). Cells were plated at 5×10^4^ cells/ml on discs for 4, 7, 10 and 14 days, respectively. At the predetermined time point, cultures were processed according to the kit instructions. ALP activity was calculated from a standard absorbance curve at 520 nm using an ELISA plate reader.

### Statistical analysis

Deviation analysis of data was performed using one-way analysis of variance (ANOVA) with a *post hoc* test. Statistical analysis was performed using SPSS 13.0. Differences and parameters were considered statistically significant at α 0.05 level.

## Results

### Pore structure, porosity and mean pore size

**Fig 1:**
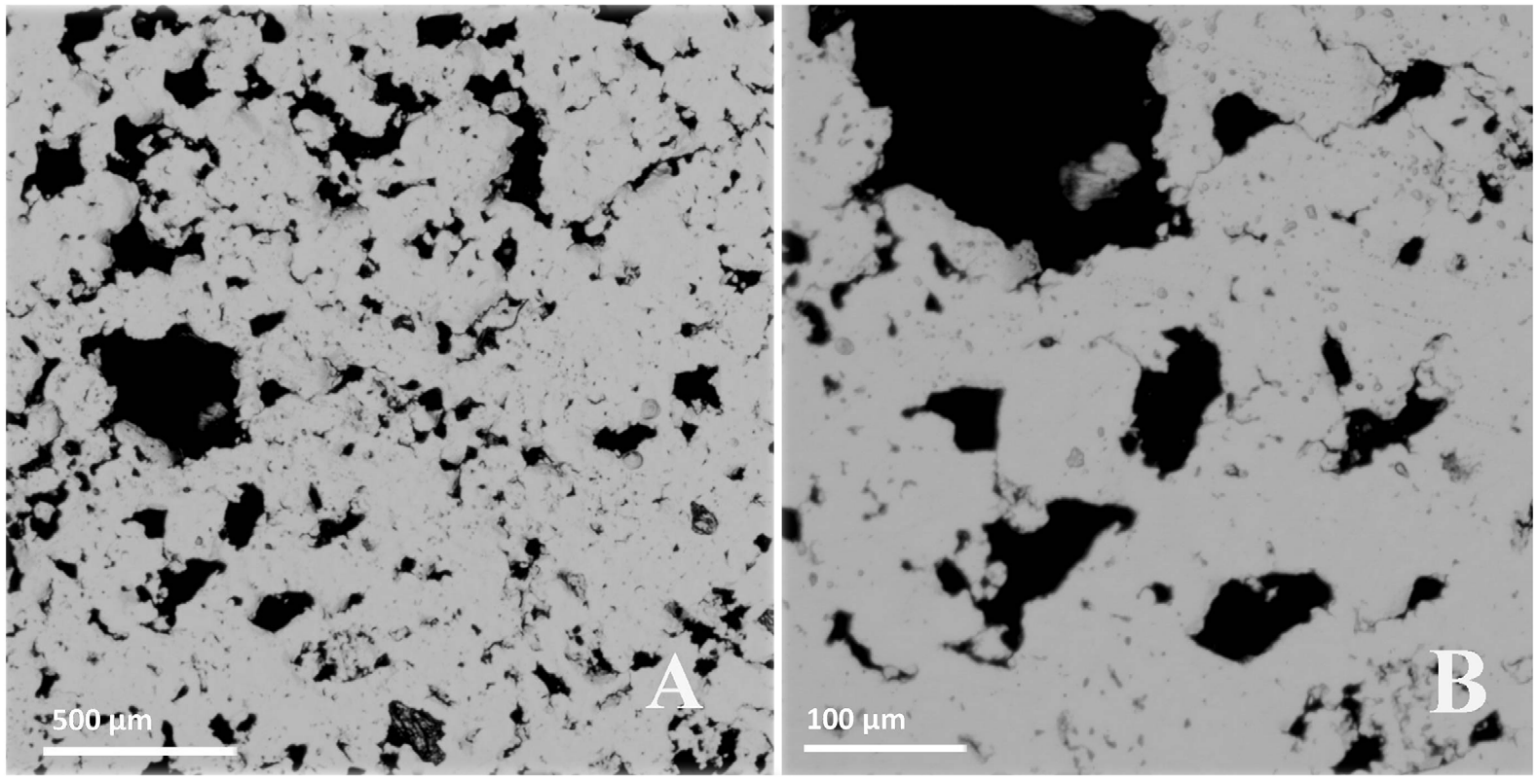
Surface display under metallographic microscope (A: 10×, B: 50×).

Porous NiTi alloy samples show a three-dimensional pore structure. Most pores have open cell structure and few isolated pores can also be observed (Fig 1). Results also show that porous NiTi alloy samples possess different mean pore sizes (MPS) and porosities after using space-holder NH_4_HCO_3_ powder with different sizes (Table 2).

**Table 2:** Porosity and Mean pore size of porous NiTi alloy samples.

Group	NH_4_HCO_3_ size (μm)	Porosity (%)	Mean pore size, *x* ± *se* (μm)
A	600 ~ 900	55.1	1013.7 ± 12.9
B	450 ~ 600	55.6	644.6 ± 18.0
C	300 ~ 450	54.2	566.5 ± 18.3
D	200 ~ 300	53.0	337.6 ± 11.0
E	50 ~ 200	53.6	277.2 ± 12.4

The Mean pore size of samples was measured by image analysis. From group A to E the MPS decreases with the descending size of the added NH_4_HCO_3_.

### Mechanical properties

All porous NiTi alloy samples in groups A to E show remarkably lower Young’s modulus than cpTi samples, as shown in Table 3. The compressive strength increases from 56.2 MPa to 108.8 MPa and fracture strength increases from 41.6 MPa to 64.6 MPa with decreasing pore size. cpTi sample, as expected, shows the highest value of both compressive strength and fracture strength (*p* < 0.05), as indicated in Table 3.

**Table 3:** Mechanical properties of porous NiTi alloy samples with different MPS.

Group	Mean pore size	Young’s modulus	Compressive strength	Fracture strength
	*x* ± *se* (μm)	*x* ± *sd* (GPa)	*x* ± *sd* (MPa)	*x* ± *sd* (MPa)
A	1013.7 ± 12.9	0.8 ± 0.2	56.3 ± 5.6	41.6 ± 6.7
B	644.6 ± 18.0	1.6 ± 0.3	68.7 ± 14.0	56.6 ± 7.5
C	566.5 ± 18.3	1.7 ± 0.2	96.6 ± 10.9	56.9 ± 4.9
D	337.6 ± 11.0	1.8 ± 0.2	108.3 ± 14.2	59.7 ± 1.8
E	277.2 ± 12.4	2.0 ± 0.2	108.8 ± 7.7	64.6 ± 2.8
F	-	112.6 ± 0.9	630.0 ± 51.3	554.8 ± 25.2

Group A ~ E are test groups, Group F (pure Ti) control group. From Group A to E samples become denser with the descending Mean pore size, and their Young’s modulus, Compressive strength as well as Fracture strength rise correspondingly.

### Cytocompatibility

**Fig 2:**
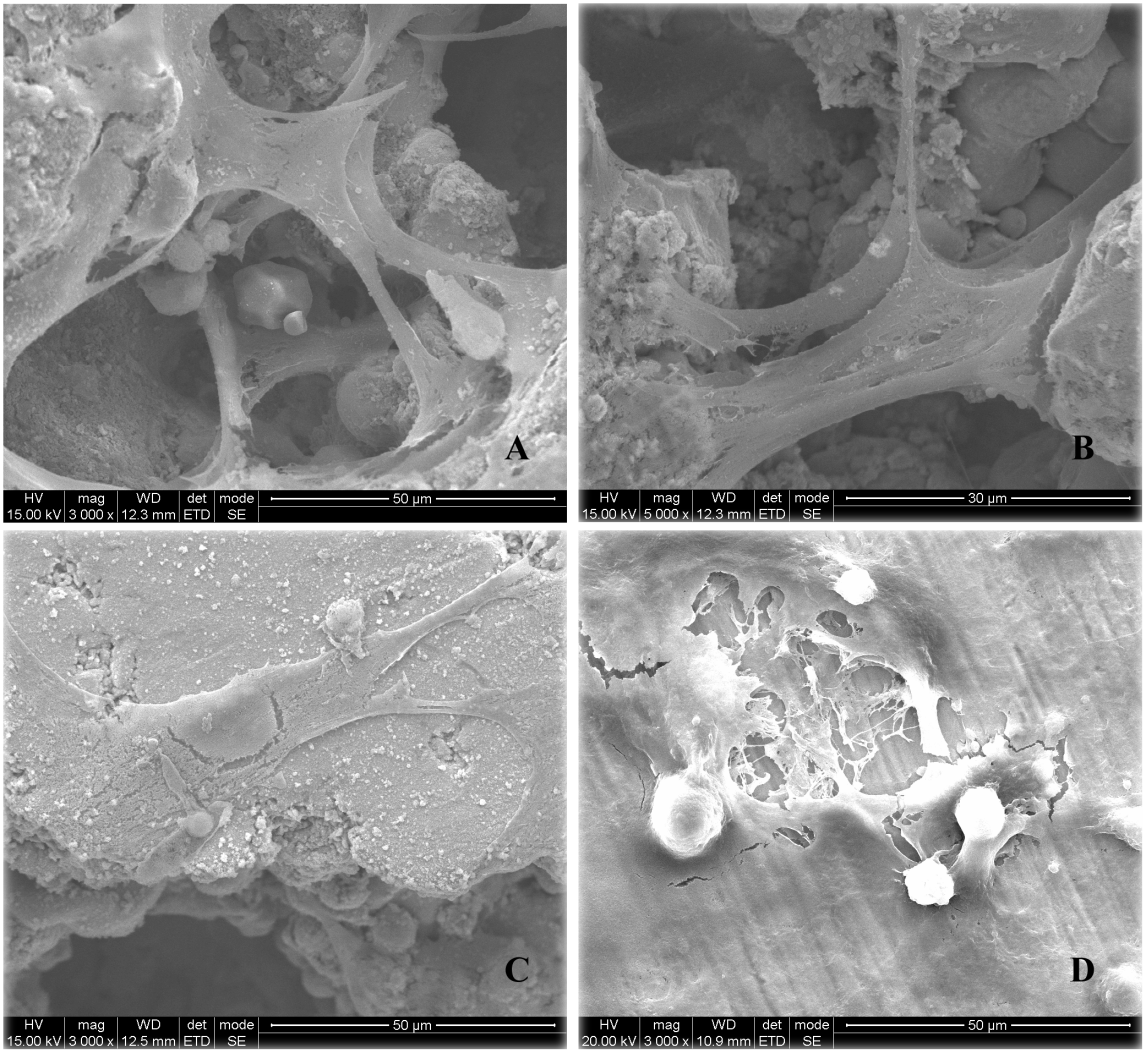
The growth of hFOB 1.19 cells on the porous NiTi alloys with different MPS of 277.2 μm (A), 337.6 μm (B) and 566.5 μm (C), and on cpTi (D).

Cells grow well on all samples, but spread much better on the surface of porous NiTi alloy samples (Fig 2A to 2C) than on cpTi (Fig 2D). The attached cells on the porous samples have flat and well-spread shapes, and also show plentiful cellular micro-extensions on porous NiTi alloy samples.

Cells migrate into the pores and span in the pores in porous samples Group E, D with MPS 277.2 μm (Fig 2A) and 337.6 μm (Fig 2B). Cells adhere fully to the exposed surface of the pores of Group D and E, with abundant extension of filopodia. However, cells seem to be “incapable” of spanning and bridging the big pores, when MPS of the sample reaches at 566.5 μm (Fig 2C).

**Fig 3:**
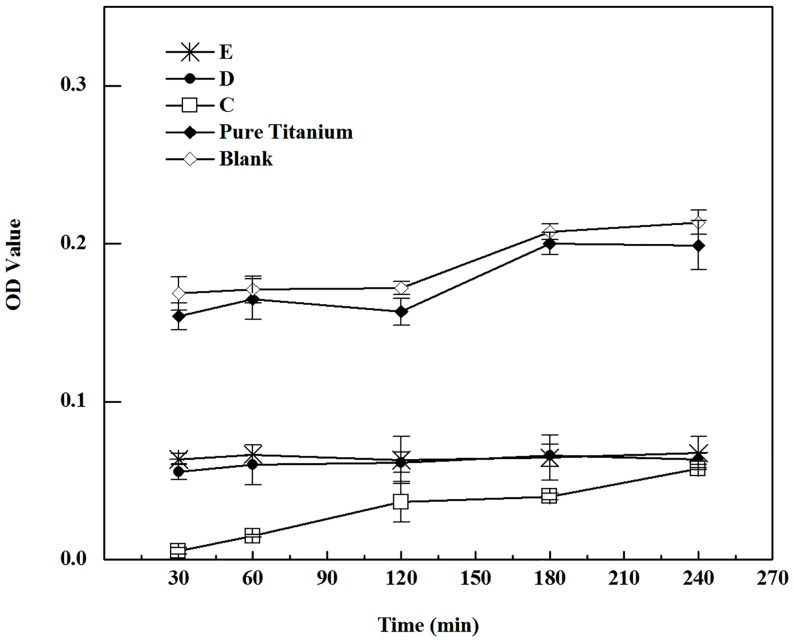
Cell adhesion on the test samples (Group C ~ E refer to samples’ MPS of 566.5 μm, 337.6 μm and 277.2 μm, respectively. Higher OD value indicates much more cells adhered on the samples).

Cell adhesion on the samples can be divided into two subgroups: C, D, E; as well as cpTi, blank. Firstly, porous samples with lower MPS have shown better cell adhesion in each time point. Secondly, the curves of both subgroups run almost ascending over time. Cell adhesion on the present sample, regardless of its surface condition, is accordingly time positively correlated (Fig 3).

**Fig 4:**
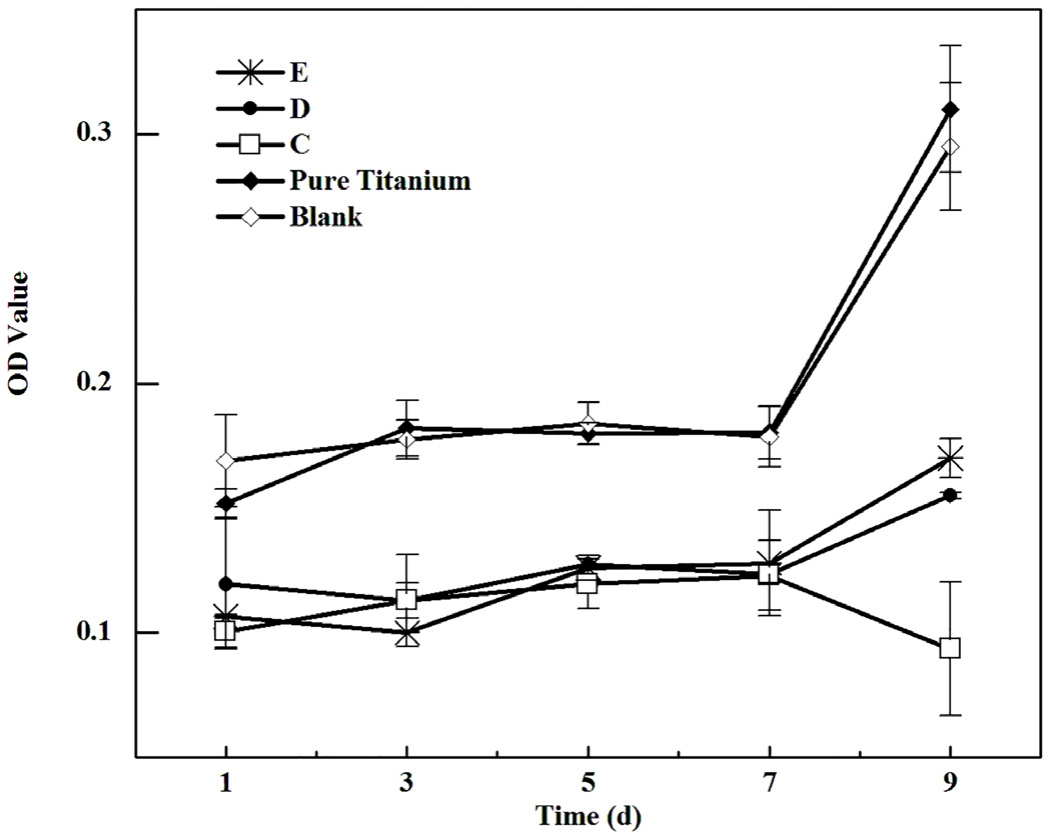
Cell growth on the test samples (Group C ~ E refer to samples’ MPS of 566.5 μm, 337.6 μm and 277.2 μm, respectively. Higher OD value indicates much more cells grew or maintained “alive” on the samples).

Cell growth on the samples can also be divided into two subgroups: C, D, E; as well as cpTi, blank. Cell growth curves proceed firstly plateau, and then reach a growing peak at day 9 on all samples except the porous NiTi alloys with the largest MPS; on the latter cells decrease after 7 days cultured. On the porous sample with smaller MPS, cells grow, in terms of viable cell numbers, better than larger MPS, though not clearly in each time point (Fig 4). Taking both cpTi sample and blank sample into consideration, cells seem to better adapt themselves on the samples with the smallest MPS, if the dense sample is regarded as the “porous” sample with a zero MPS.

**Fig 5:**
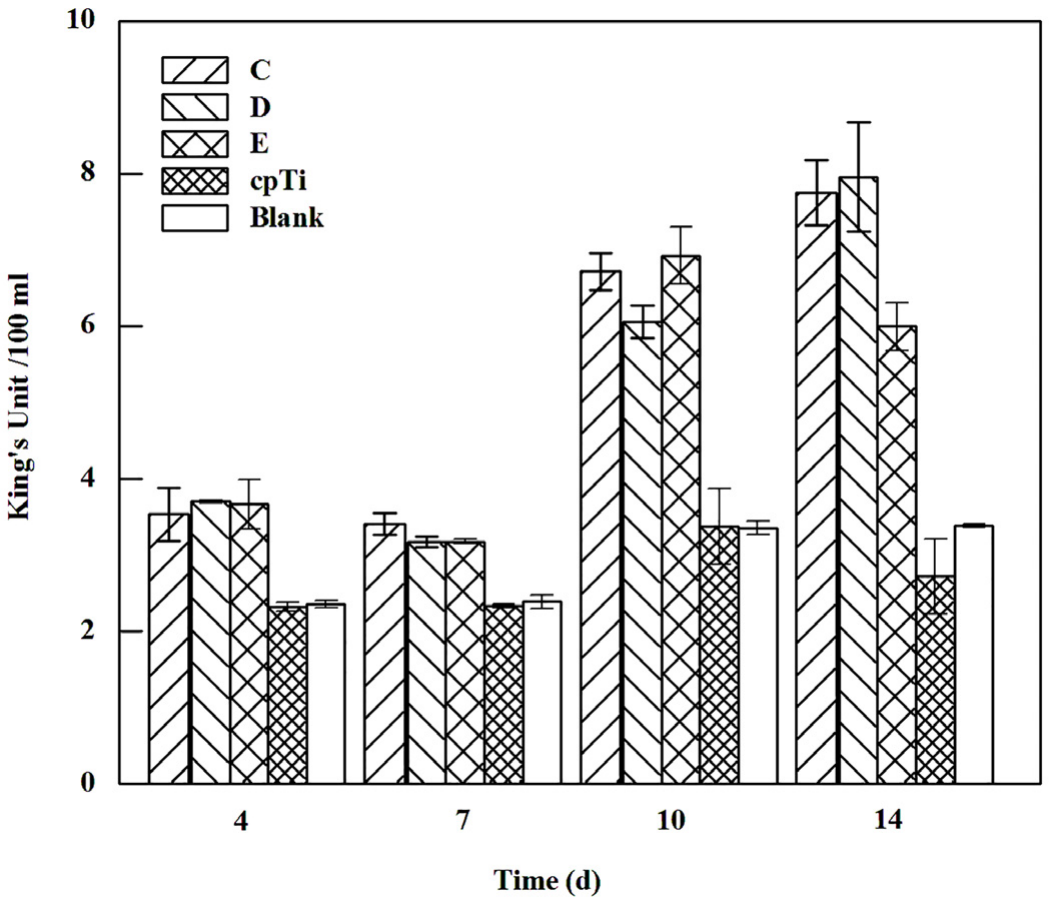
ALP activity on porous NiTi alloys and the control samples (Group C ~ E refer to samples’ MPS of 566.5 μm, 337.6 μm and 277.2 μm, respectively).

ALP activity rises with time and reaches a peak at day 14. Cell ALP activity in all porous samples of three groups is higher than on the control group at each time point (*p* < 0.05). No remarkable change in tendency of ALP activity is observed within three porous groups (Fig 5). The ALP activity of cells on the porous samples appears not to be related to their MPS.

## Discussion

In the present study, porous NiTi alloy samples were prepared by using pore-forming process with powder metallurgy method, in which NH_4_HCO_3_ is employed as a temporary space-holder and porosity regulator. With the aid of NH_4_HCO_3_ powder of different particles sizes, the MPS of porous samples is controlled ranging from 277.2 to 1013.7 μm, and the porosity of each group is kept in a limited range (53.0% ~ 55.6%). This indicates that porous structure can be introduced into the dense NiTi alloys by means of using pore-forming agent NH_4_HCO_3_, in comparison with other method [20], and the MPS and porosity of porous NiTi samples can be regulated separately, and modified by changing the particle size of NH_4_HCO_3_ powder.

Among the open cell structure mainly in the samples, very few isolated pores were observed. These secondary pores have been attributable to the trapped residuals of NH_4_HCO_3_ decomposition and/or the non-metallic impurities existing in the raw powders [15]. The isolated pores would co-contribute, together with the open cell structure, to the reduction of Young’s modulus of NiTi alloy. The isolated pores on the superficial layer of the samples would supply several osteoblasts with tiny space for the bony ingrowth. Taking the negligible total amount of isolated pores in consideration, however, their influence on the biomechanical properties of porous NiTi, in comparison to the open pores, is suggested to be much less evident.

The elastic modulus of such developed porous NiTi alloy samples should be decreased accordingly to fit the nature bone. Generally, the elastic modulus of human cancellous bone is not higher than 3 GPa, and cortical bone 12 GPa [21]. Our results showed that the elastic modulus of all porous NiTi alloy samples is in the range of 0.8 to 2.0 GPa, very close to that of cancellous bone. A good match of Young’s modulus between the porous NiTi alloy and natural bone may lead to an enhanced load transfer to the surrounding bone.

The longevity of the *in-vivo* use depends not only on the elastic modulus, but also on other mechanical properties such as the compressive strength and fracture strength. Clearly, the compressive strength of the present porous NiTi alloy samples is controlled to be much lower than that of the cpTi after the porous structure is introduced. Compared with the compressive strength of human mandible cortical bone (133.6 MPa) [22], the porous NiTi alloy samples in Group E with the smallest MPS among all groups remain strength of 108.8 MPa which is close to the natural bone. However, the porous structure has led to decrease in fracture strength of porous NiTi alloy samples. Even the samples with the smallest MPS exhibit lower fracture strength value (64.6 MPa) than natural bone, e.g., 150 MPa of human femur [23]. Results indicated that the mechanical property of porous NiTi alloy samples are negatively correlated with MPS of the samples. Taking the results of elastic modulus into consideration, a smaller MPS of porous NiTi alloys, 277.2 μm for instance, might better fit the mechanical property of the natural bone system.

How to optimize the porous structure of porous NiTi alloys, in terms of increasing fracture strength on a par with natural bone, is still to be solved for the clinical application of this approach. Regardless, the introduction of porous structure can fit the elastic modulus of NiTi alloys close to the natural bone. A biomedical device may consist of a combination of dense and porous NiTi alloys. Recently, radial gradient ascending porosity towards the sample surface is processed in the porous NiTi alloys [15]. The method provides porous NiTi alloys with enhanced mechanical properties.

A balance should be sought between the mechanical properties and clinical utilization. We chose Group C, D and E with MPS of 566.5, 337.6 and 277.2 μm respectively for the tests due to their better mechanical properties displayed.

Cells adhere well to all samples tested, but have better affinity on the porous samples with MPS of 277.2 μm and 337.6 μm based on the SEM morphology, in terms of flat and well-spread shapes. Since cell morphology is closely related to the function of cells adhering to material surface [24], the present results indicated a strong cell adhesion on the porous NiTi alloys, and those with MPS of 277.2 μm and 337.6 μm are favorable for cell adhesion.

The present results clearly showed that cell adhesion and growth on porous NiTi alloy samples are both negatively correlated to MPS of samples. We have seen that it might be related to parameter of specific surface area (*S/V*). The *S/V* of porous discs is inversely proportional to the MPS at the same porosity [25], i.e., smaller pores possess a higher *S/V*. Thus, a porous material with higher *S/V* will allow more cells to attach. Our findings are in accordance with a previous study which shows that the porous Ti with a MPS of 200 μm promotes cell proliferation [9]. The distinguish cell adhesion and growth on the present porous samples also confirm a well known finding that the surface topography of a bone-substitute material has a crucial role in cell behavior and osseointegration at the interface of the bone and implant [26,27].

The impact of surface topography of a bone-substitute material on osteoblasts differentiation could be displayed through the ALP activity results. Cells show higher ALP activity on porous NiTi alloys than on cpTi, and the difference in ALP activity among porous NiTi alloy samples of different groups is almost negligible. The present results suggest that the porous structure is a major inducement, if not entirely, in ALP expression of osteoblasts growing on it, which are also consistent with the study that a porous Ti induces greater ALP activity than dense Ti in MC3T3-E1 cells [28].

## Conclusions

Cell structure can be introduced and adjusted in NiTi alloy by NH_4_HCO_3_ with different amounts and particle sizes. The mechanical properties of porous NiTi alloys are correlated negatively to the MPS. The porous structure of porous NiTi alloys is favorable to osteoblasts ingrowth, and can better promote its differentiation than cpTi.
