# Correction: Effect of Pore Size and Porosity on the Biomechanical Properties and Cytocompatibility of Porous NiTi Alloys

**DOI:** 10.1371/journal.pone.0149051

**Published:** 2016-02-05

**Authors:** Yu-Tao Jian, Yue Yang, Tian Tian, Clark Stanford, Xin-Ping Zhang, Ke Zhao

Dr. Xin-Ping Zhang should be noted as co-corresponding author with Dr. Ke Zhao. Dr. Zhang’s email address is: mexzhang@scut.edu.cn.

There are errors in the Funding section. The correct funding information is as follows: Funding for this work came from the Natural Science Foundation of China (No. 50871039, http://www.nsfc.gov.cn/) and the Guangdong Provincial Natural Science Foundation (Key Grant No. S2013020012805, http://www.gdstc.gov.cn/).
